# Property attributes influencing profitability before and after conversion of vacant long-term rentals to short-term rentals

**DOI:** 10.1371/journal.pone.0336375

**Published:** 2025-11-07

**Authors:** Kentaro Murota, Yangcheng Gu, Xiaorui Wang, Daisuke Matsushita

**Affiliations:** Department of Living Environment Design, Graduate School of Human Life and Ecology, Osaka Metropolitan University, Osaka, Japan; University of Jaen: Universidad de Jaen, SPAIN

## Abstract

In Japan, the growing number of vacant housing units has become a pressing issue, and an increase in inbound tourism has led to accommodation shortages and rising lodging prices. Converting long-term rentals (LTRs) into short-term rentals (STRs) has emerged as a practical solution; however, empirical evidence on which property attributes are best suited for such conversions remains limited. This study identified the physical and locational attributes of vacant LTRs in Tokyo’s 23 wards, which contributed to improved profitability following conversion. Using an open dataset of 7,772 Airbnb listings from 2024, we developed a hedonic pricing model to estimate rates based on floor area, distance to the nearest station, station ridership, nearby tourist attractions, and administrative districts. The model was applied to 20 930 LTR properties to estimate the profit ratio before and after conversion and identify favorable attributes. Higher STR profitability was linked to lower pre-conversion rent, proximity to high-ridership stations, and locations near many tourist attractions within 10 km, especially in wards near transport hubs (e.g., Setagaya, Shibuya, Meguro, Shinagawa, and Shinjuku) and in well-connected, lower-rent peripheral wards (e.g., Nakano and Suginami). These findings offer insights into housing stock utilization and provide guidance for STR regulations and tourism policies.

## Introduction

The increasing number of vacant housing units in Japan has become a serious social issue. Japan faces a serious social issue: the rapid rise in vacant housing units. Owing to population decline and the concentration of residents in major urban areas, the effective use of housing stock, particularly in rural regions, has been increasingly emphasized [1]. Even in urban areas, many housing units with poor building conditions are left vacant, as some units are considered non-rebuildable properties due to inadequate access to roads or inaccessibility by vehicle [2]. Despite new legal frameworks such as the Act on Special Measures for Promotion of Countermeasures against Vacant Houses, the number of vacant homes continues to increase [3]. While housing supply continues to increase in areas with low demand, accommodation shortages are emerging due to rising inbound tourism. In Japan, the recent surge in international tourists has caused a shortage of accommodations and an urgent need for the development of alternative lodging options [4]. Simultaneously, the growth of the sharing economy—facilitated by advances in information technology—has highlighted new forms of real estate utilization that differ from conventional long-term rental (LTR) housing [5]. Airbnb, the home-sharing platform, entered the Japanese market in 2013 and has grown rapidly. According to Oxford Economics, the Airbnb community contributed approximately ¥405.5 billion (USD 2.7 billion) to Japan’s GDP and supported approximately 41,500 jobs in 2022, indicating the significant economic and tourism-related impact of short-term rentals (STRs) in Japan [6,7]. A U.S. study further reported that STRs could stimulate housing investments [8].

Despite these positive effects, concerns about STRs have arisen. Airbnb’s expansion has been linked to gentrification [9–11] and overtourism [12] in urban areas, prompting regulatory interventions. In Barcelona, unauthorized Airbnb hosts are subject to severe penalties [13], and Amsterdam has implemented restrictions on the number of rental days to protect its housing market [14]. Ayouba et al. (2020) found that STRs have contributed to upward pressure on rents in certain French cities [15], whereas studies in the U.S. suggest that the effects on housing prices and rents may be more neutral [16]. Balancing the promotion and regulation of STRs has become a key issue in tourism policies.

The vacancy issue is largely due to the mismatch between supply and demand in the rental housing market [17]. Although LTRs and STRs involve rental transactions, the required property attributes differ. STR guests are often travelers, especially international tourists, who may not value the long-term residential quality or vehicle accessibility of a property. Unlike long-term residents, they are less likely to prioritize insulation, energy efficiency, safety, and quiet residential environments. Instead, they tend to prefer accessible locations, proximity to commercial amenities, unique residential experiences, and local cultural features [18,19]. Therefore, vacant LTRs that fail to meet current market demands may nevertheless align with the preferences of STR guests. Conversely, not all LTRs are suitable for conversion. Although real estate professionals and investors may have practical insights, empirical evidence on which property attributes enhance profitability when converting LTRs into STRs remains limited. Although previous studies have examined the factors determining Airbnb prices in Western cities [20–25], few have focused on Asian countries, including Japan [26]. Japan-focused research can help supplement the existing literature because its contextual conditions differ significantly from those in Western countries.

To investigate the change in profitability from converting vacant LTRs into STRs, we developed a hedonic pricing model using open datasets for rental apartments and STRs. A hedonic pricing model is a regression-based approach that quantifies the contribution of different property attributes to the price [27]. By applying this model to a large-scale dataset, we aim to identify the contribution of physical and locational property attributes to profitability before and after conversion. This study focuses on Tokyo’s 23 wards and aims to clarify how property attributes affect profitability when converting vacant LTRs into STRs. By comparing the contributions of the attributes to both LTR rent and STR revenue, we aim to identify the characteristics that are more effective for each market. These findings provide evidence for housing stock utilization, accommodation supply policies, and rational STR regulations.

## Materials and methods

The data used in this study were obtained entirely from publicly available sources (Inside Airbnb, National Land Numerical Information, and At Home Dataset).

The collection and analysis of these data complied with the terms and conditions of the respective data providers.

### Study design

**Fig 1:**
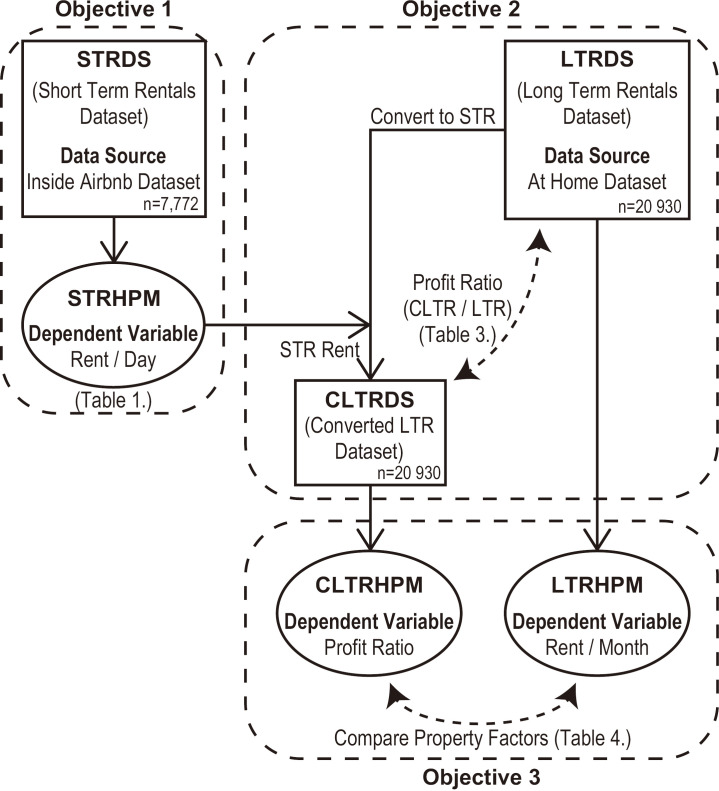
The relationship between the three objectives. STRDS: Short-term rental datasets; STRHPM: Short-term rental hedonic pricing model; LTRDS: Long-term rental datasets; LTRHPM: Long-term rental hedonic pricing model; CLTRDS: Converted long-term rental datasets; CLTRHPM: Converted Long-term rental hedonic pricing model.

As shown in Fig 1, this study consists of three sub-objectives.

In Objective 1, we constructed a hedonic pricing model (STRHPM: Short-Term Rental Hedonic Pricing Model) to estimate nightly rates for STRs in Tokyo’s 23 wards. This was based on an open dataset of STR listings (STRDS: Short-Term Rental Dataset). The model is summarized in Table 1. The dependent variable was the nightly rate, and the independent variables included the estimated floor area, straight-line distance to the nearest station, daily station ridership, number of tourist attractions within 1 km, 5 km, and 10 km radius, and administrative district.

**Table 1:** Linear regression model for estimating STR profitability (STRHPM).

Independent variable	Variable	Coefficient	*SE*	*t*	Standardized β	95%CI		*p*
						*LL*	*UL*	
Intercept		1774.6869	578.0404	3.07	0.000	641.57317	2907.8007	0.0021
Estimated floor area (${\text{m}}^{\text{2}}$)	*EA*	211.54839	3.294285	64.22	0.560	205.09071	218.00607	<.0001
Distance to the nearest station (m)	*DS*	−2.75672	0.33452	−8.24	−0.075	−3.412469	−2.10097	<.0001
Nearest station’s ridership (person/day)	*PS*	0.0068412	0.000882	7.76	0.069	0.005112	0.0085704	<.0001
Tourist attraction								
Within 1 km	*SS1*	970.44168	102.6775	9.45	0.104	769.16643	1171.7169	<.0001
Within 5 km	*SS5*	61.107031	23.61098	2.59	0.050	14.823202	107.39086	0.0097
Within 10 km	*SS10*	94.892881	20.12703	4.71	0.116	55.43852	134.34724	<.0001
Administrative district	*AD*							
Chiyoda		4015.0689	1150.052	3.49	0.039	1760.6603	6269.4774	0.0005
Chuo		4040.1766	896.4065	4.51	0.069	2282.9803	5797.373	<.0001
Minato		4586.1711	760.6201	6.03	0.131	3095.1523	6077.1898	<.0001
Shinjuku		1895.9591	694.2433	2.73	0.113	535.0568	3256.8615	0.0063
Bunkyo		−1150.385	835.6872	−1.38	−0.023	−2788.556	487.78489	0.1687
Taito		678.71813	727.4016	0.93	0.027	−747.1835	2104.6197	0.3508
Sumida		−1295.818	669.5896	−1.94	−0.062	−2608.393	16.756593	0.053
Koto		−859.6789	796.6693	−1.08	−0.016	−2421.364	702.00583	0.2806
Shinagawa		2511.7018	835.7482	3.01	0.039	873.41206	4149.9916	0.0027
Meguro		4083.9813	1089.3	3.75	0.038	1948.6618	6219.3009	0.0002
Ota		114.11397	602.8994	0.19	0.003	−1067.73	1295.9579	0.8499
Setagaya		3454.7915	625.14	5.53	0.095	2229.3501	4680.2329	<.0001
Shibuya		5561.8035	667.7867	8.33	0.199	4252.7632	6870.8439	<.0001
Nakano		1985.5953	655.9247	3.03	0.052	699.80761	3271.383	0.0025
Suginami		1989.1897	664.3854	2.99	0.043	686.81675	3291.5627	0.0028
Teshima		754.74383	729.3599	1.03	0.033	−674.9966	2184.4842	0.3008
Kita		−1362.456	751.9052	−1.81	−0.030	−2836.391	111.47943	0.07
Arakawa		−1602.456	998.4981	−1.6	−0.019	−3559.779	354.86731	0.1086
Itabashi		143.59173	727.387	0.2	0.003	−1282.281	1569.4646	0.8435
Adachi		−502.5809	900.9721	−0.56	−0.006	−2268.727	1263.5652	0.577
Edogawa		308.87882	714.2132	0.43	0.006	−1091.17	1708.9276	0.6654
Katsushika		−2083.433	755.1816	−2.76	−0.035	−3563.79	−603.0747	0.0058

**Table 2:** Cleaning fees by STR floor area.

Floor Area (${\text{m}}^{\text{2}}$)	Cleaning fee (JPY)
15–25	5,000
25–30	5,500
30–40	6,000
40–50	6,500
50–60	7,000
60–70	7,500
70–80	8,000
80–90	8,500
90–100	9,000
100–150	9,500

In Objective 2, we applied the STRHPM to all properties listed in an open dataset of vacant LTRs in Tokyo’s 23 wards (LTRDS: Long-Term Rental Dataset) to estimate the expected nightly rates after conversion to STRs. Consequently, we constructed a dataset of converted LTRs (CLTRDS: Converted Long-Term Rental Dataset). We then calculated the expected monthly STR revenue and profitability ratio (PR)—the ratio of STR revenue to pre-conversion LTR rent. A binary logistic regression model was used to examine which property attributes were associated with PR ≥ 1, and odds ratios were calculated for each attribute category (Table 3).

**Table 3:** Bivariate logistic regression analysis of profitability ratio ≥ 1 under three assumed occupancy rates.

	Model1: Occupancy rate 80%		Model2: Occupancy rate 70%		Model3: Occupancy rate 60%	
	Unadjusted odds ratio (95% CI)	*p*	Unadjusted odds ratio (95% CI)	*p*	Unadjusted odds ratio (95% CI)	*p*
**Rent (JPY/month)**						
<¥100,000	1(reference)		1(reference)		1(reference)	
¥100,000 – ¥200,000	0.95(0.85–1.05)	0.308	0.52(0.45–0.59)	<.0001	0.29(0.22–0.37)	<.0001
¥200,000 – ¥300,000	0.42(0.22–0.81)	0.010	0.05(0.01–0.38)	0.004	0.00(0.00–.)	0.976
¥300,000 <	0.21(0.03–1.76)	0.211	0.00(0.00–.)	0.977	0.00(0.00–.)	0.991
**Floor area (**${\text{m}}^{\text{2}}$)						
<35(${\text{m}}^{\text{2}}$)	1(reference)		1(reference)		1(reference)	
35 m² – 55m²	0.81(0.75–0.88)	<.0001	0.50(0.45–0.55)	<.0001	0.32(0.27–0.38)	<.0001
55m² – 75m²	1.09(0.86–1.39)	0.458	0.80(0.61–1.05)	0.105	0.44(0.27–0.70)	0.001
75m² – 95m²	1.18(0.62–2.25)	0.618	0.55(0.24–1.25)	0.153	0.56(0.17–1.82)	0.334
95m² <	2.70(1.02–7.09)	0.026	1.37(0.54–3.49)	0.506	1.19(0.35–4.08)	0.785
<500m	1(reference)		1(reference)		1(reference)	
500m – 1,000m	0.38(0.35–0.40)	<.0001	0.33(0.31–0.36)	<.0001	0.33(0.30–0.36)	<.0001
1,000m – 1,500m	0.12(0.10–0.14)	<.0001	0.07(0.06–0.09)	<.0001	0.04(0.02–0.02)	<.0001
1,500m <	0.02(0.01–0.04)	<.0001	0.01(0.00–0.04)	<.0001	0.00(0.00–.)	0.974
> 100,000	1(reference)		1(reference)		1(reference)	
100,000–200,000	0.90(0.82–0.99)	0.026	1.26(1.14–1.39)	<.0001	1.66(1.46–1.89)	<.0001
200,000–300,000	13.80(9.59–19.85)	<.0001	14.60(11.03–19.34)	<.0001	15.06(12.10–18.74)	<.0001
300,000 <	3.13(2.44–4.01)	<.0001	3.45(2.74–4.34)	<.0001	4.63(3.64–5.90)	<.0001
Yes	1(reference)		1(reference)		1(reference)	
No	0.08(0.05–0.11)	<.0001	0.09(0.06–0.12)	<.0001	0.07(0.05–0.09)	<.0001
< 5	1(reference)		1(reference)		1(reference)	
5–10	3.37(3.06–3.71)	<.0001	3.80(3.47–4.16)	<.0001	5.81(5.24–6.44)	<.0001
10–15	1.91(1.61–2.26)	<.0001	2.82(2.38–3.33)	<.0001	5.16(4.30–6.20)	<.0001
15–20	2.72(2.06–3.59)	<.0001	3.50(2.68–4.57)	<.0001	5.21(3.91–6.94)	<.0001
20 <	12.69(4.50–35.78)	<.0001	6.92(3.49-13.73)	<.0001	10.71(5.66–20.28)	<.0001
< 5	1(reference)		1(reference)		1(reference)	
5–10	2.66(2.47–2.87)	<.0001	2.93(2.67-3.22)	<.0001	3.45(2.94–4.04)	<.0001
10–15	3.36(3.08–3.67)	<.0001	3.47(3.13-3.85)	<.0001	4.01(3.37–4.75)	<.0001
15–20	4.32(3.85–4.85)	<.0001	4.79(4.22-5.43)	<.0001	7.62(4.34–9.16)	<.0001
20–25	5.64(5.04–5.31)	<.0001	8.05(5.15-9.07)	<.0001	13.82(5.69–16.35)	<.0001
25 <	5.56(4.96–6.22)	<.0001	7.14(6.33-8.05)	<.0001	12.16(6.26–14.43)	<.0001
**Administrative district**						
Nerima	1(reference)		1(reference)		1(reference)	
Chuo	1883970.5(0.00–3.25E + 304)	0.967	3796157.70(1.00–6.55E + 304)	0.966	78.95(10.19–611.78)	<.0001
Minato	9.60(5.11–18.02)	<.0001	10.13(1.11–16.79)	<.0001	13.28(8.67–20.35)	<.0001
Shinjuku	15.29(11.59–20.17)	<.0001	8.08(1.78–9.64)	<.0001	5.72(4.88–6.71)	<.0001
Bunkyo	0.31(0.24–0.42)	<.0001	0.17(1.10–0.27)	<.0001	0.15(0.07–0.33)	<.0001
Taito	13.05(6.00–28.38)	<.0001	12.99(1.16–23.58)	<.0001	9.90(6.31–15.55)	<.0001
Sumida	0.29(0.19–0.45)	<.0001	0.33(1.19–0.56)	<.0001	0.46(0.22–0.93)	0.032
Koto	0.18(0.12–0.27)	<.0001	0.09(1.04–0.19)	<.0001	0.14(0.05–0.37)	<.0001
Shinagawa	10.00(7.80–12.80)	<.0001	3.78(1.21–4.46)	<.0001	1.86(1.52–2.28)	<.0001
Meguro	20.57(15.60–27.14)	<.0001	10.95(1.26–12.96)	<.0001	5.98(5.19–6.90)	<.0001
Ota	0.02(0.01–0.02)	<.0001	0.01(1.00–0.02)	<.0001	0.00(0.00–0.03)	<.0001
Setagaya	8.08(7.46–8.75)	<.0001	4.79(1.47–5.14)	<.0001	2.35(2.15–2.56)	<.0001
Shibuya	88.30(41.84–186.36)	<.0001	53.63(1.23–81.65)	<.0001	60.98(46.06–80.74)	<.0001
Nakano	1.86(1.65–2.09)	<.0001	0.95(1.83–1.08)	0.423	0.47(0.38–0.60)	<.0001
Suginami	0.48(0.44–0.53)	<.0001	0.23(1.20–0.26)	<.0001	0.11(0.08–0.14)	<.0001
Teshima	2.40(2.08–2.77)	<.0001	1.64(1.43–1.89)	<.0001	1.27(1.05–1.54)	0.014
Kita	0.08(0.06–0.10)	<.0001	0.05(1.03–0.08)	<.0001	0.05(0.02–0.12)	<.0001
Arakawa	0.28(0.20–0.39)	<.0001	0.15(1.08–0.26)	<.0001	0.06(0.02–0.26)	0.0001
Itabashi	0.11(0.09–0.13)	<.0001	0.06(1.04–0.09)	<.0001	0.04(0.02–0.09)	<.0001
Adachi	0.40(0.35–0.46)	<.0001	0.28(1.23–0.33)	<.0001	0.18(0.13–0.26)	<.0001
Edogawa	0.11(0.09–0.13)	<.0001	0.05(1.04–0.07)	<.0001	0.02(0.01–0.04)	<.0001
Katsushika	0.02(0.01–0.04)	<.0001	0.02(1.01–0.04)	<.0001	0.01(0.001–0.059)	<.0001

In Objective 3, we developed a hedonic pricing model to estimate the monthly rent for vacant LTRs (LTRHPM: Long-Term Rental Hedonic Pricing Model) and another to estimate PRs after conversion (CLTRHPM: Converted Long-Term Rental Hedonic Pricing Model). By comparing the standardized regression coefficients and contribution rates of both models, we identified the attributes that contributed to profitability in the STRs and LTRs (Table 4).

**Table 4:** Linear regression models: LTRHPM for estimating long-term rental profitability and CLTRHPM for estimating profitability ratios before and after STR conversion.

	LTRHPM (before conversion)					CLTRHPM (after conversion)				
Independent variable	Coefficient	Standardized β	Contribution rate (*CR*)	*p*	Adjusted power (0.05)	Coefficient	Standardized β	Contribution rate (*CR*)	*p*	Adjusted power (0.05)
Floor are (${\text{m}}^{\text{2}}$)	1718.03	0.824	80.00%	<.0001	1.0	0.0076	0.206	5.32%	<.0001	1.0
Distance to the nearest station (m)	−6.93	−0.111	1.45%	<.0001	1.0	−0.0004	−0.355	15.79%	<.0001	1.0
Nearest station ridership (person/day)	0.015	0.043	0.22%	<.0001	1.0	0.000001	0.155	3.01%	<.0001	1.0
Number of Tourist attraction										
Within 1 km	4467.76	0.024	0.07%	<.0001	1.0	0.13	0.04	0.20%	<.0001	1.0
Within 5 km	369	0.054	0.34%	<.0001	1.0	0.00235	0.02	0.05%	0.0025	0.82
Within 10 km	318	0.125	1.84%	<.0001	1.0	0.013	0.302	11.43%	<.0001	1.0
Administrative ward										
Chuo	7384.09	0.008	0.01%	0.03	0.50	0.44	0.029	0.11%	<.0001	1.0
Minato	31856.26	0.097	1.11%	<.0001	1.0	0.19	0.035	0.15%	<.0001	1.0
Shinjuku	4900.63	0.04	0.19%	<.0001	1.0	0.19	0.093	1.08%	<.0001	1.0
Bunkyo	2880.73	0.015	0.03%	<.0001	0.86	−0.26	−0.085	0.91%	<.0001	1.0
Taito	−1905.79	−0.005	0.00%	0.21	0.12	0.16	0.027	0.09%	<.0001	1.0
Sumida	−3340.71	−0.012	0.02%	<.0001	0.83	−0.22	−0.047	0.28%	<.0001	1.0
Koto	2335.9	0.011	0.01%	0.01	0.69	−0.21	−0.058	0.42%	<.0001	1.0
Shinagawa	9541.69	0.073	0.63%	<.0001	1.0	0.23	0.104	1.36%	<.0001	1.0
Meguro	15550.22	0.141	2.34%	<.0001	1.0	0.41	0.223	6.23%	<.0001	1.0
Ota	9755.7	0.122	1.75%	<.0001	1.0	−0.15	−0.111	1.54%	<.0001	1.0
Setagaya	10265.62	0.196	4.53%	<.0001	1.0	0.42	0.472	27.91%	<.0001	1.0
Shibuya	14765.28	0.1	1.18%	<.0001	1.0	0.62	0.252	7.96%	<.0001	1.0
Nakano	5900.75	0.064	0.48%	<.0001	1.0	0.22	0.143	2.56%	<.0001	1.0
Suginami	9136.1857	0.143	2.41%	<.0001	1.0	0.18	0.171	3.66%	<.0001	1.0
Teshima	2174.0796	0.02	0.05%	<.0001	0.97	0.042	0.023	0.07%	<.0001	0.99
Kita	1740.0681	0.015	0.03%	0.001	0.88	−0.33	−0.169	3.58%	<.0001	1.0
Arakawa	−4275.387	−0.02	0.05%	<.0001	1.0	−0.27	−0.075	0.70%	<.0001	1.0
Itabashi	164.1882	0.002	0.00%	0.65	0.05	−0.072	−0.049	0.30%	<.0001	1.0
Adachi	−8823.696	−0.094	1.04%	<.0001	1.0	−0.052	−0.033	0.14%	<.0001	1.0
Katsushika	−4883.507	−0.044	0.23%	<.0001	1.0	−0.38	−0.203	5.16%	<.0001	1.0

*Note.* LTRHPM Adjusted R^2^: 0.71, CLTRHPM Adjusted R^2^: 0.77

## Procedure

### Objective 1: Construction of the STR Hedonic Pricing Model (STRHPM)

To create the STRDS, we used an open dataset published by Inside Airbnb, an independent project that scrapes, analyzes, and visualizes Airbnb listing data [28]. The most recent dataset version as of September 5, 2024, was used and included 16,841 Airbnb listings in Tokyo. In addition to host information, such as listing ID and name, the dataset contained property attributes, including latitude, longitude, maximum occupancy, number of bathrooms and bedrooms, nightly rate, and review score. The dataset is not officially provided by Airbnb, but has been widely used in prior studies and is recognized as a valuable source for research and policy [29]. First, we removed 1,884 listings with missing or erroneous data. Then, we filtered for LTR-type properties by selecting listings with ‘entire rental unit’ or ‘entire serviced apartment’ as the property type, and ‘entire home/apt’ as the room type; this resulted in 9,306 listings. To avoid the duplication of identical units in the same building, we excluded 1,170 listings with the same coordinates and occupancy. Additionally, we excluded 284 listings whose Z-score for nightly rate exceeded ±2, treating them as outliers [30]. The final sample comprised 7,772 listings.

As the STRDS does not include floor area, we estimated the floor area. The Ministry of Land, Infrastructure, Transport and Tourism sets a minimum housing standards [31] based on the number of tenants, and all households aim to meet this level (Equation 1). Since STR tends to set the maximum number of guests in order to maximize profits, we estimated floor area from the minimum housing standards. We estimated floor area by using the maximum occupancy of Airbnb listing data [28] with the number of tenants in Equation 1.

For NT = 1: GFA = 25


For NT ≥ 2: GFA = 10 × NT + 10 (1)

where NT: Number of tenants,

GFA: Gross floor area (m²).

We also derived additional locational attributes using GIS and publicly available data from the National Land Numerical Information Database.

Distance to the nearest station (m) [32]

Station ridership (passengers/day) [33]

Number of tourist attractions within 1 km, 5 km, and 10 km [34]

Using the STRDS of 7,772 listings, we constructed the STRHPM to estimate nightly rates based on property characteristics. The linear regression model is as follows:


$$NR=\alpha +\Sigma \beta_{n}X_{n}+\;\varepsilon$$ (2)

where NR = nightly rate (JPY/day),

*α* = intercept,

*β*_*n*_ = parameter vector,

*X*_*n*_ = matrix of property attributes,

*ε* = error term.

The dependent variable was nightly rate (JPY/day), and the independent variables were estimated floor area (m²), distance to the nearest station (m), station ridership (passengers/day), number of tourist attractions within 1 km, 5 km, and 10 km, and administrative district. To avoid dummy variable traps, Nerima Ward was omitted as a reference category [35].

### Objective 2: Construction of LTRDS and CLTRDS and Estimation of profitability ratios

To construct the LTRDS, we used the ‘At Home Dataset,’ provided by At Home Co., Ltd. via the National Institute of Informatics (NII) [36]. This dataset includes property information registered in a nationwide real estate network and covers variables such as rent, price, property specifications (floor area, layout, structure, and construction year), location (address, nearest rail line and station, walking time, and coordinates), and facilities. The dataset contains information on properties listed from January 1, 2018, to December 31, 2022, with approximately 4–5 million listings per year. We used the data from 2022 for rental apartments in Tokyo (229,290 listings) and filtered these to include only properties located within the 23 wards (147,101 listings). To remove the duplicates of identical units in the same building, listings with identical coordinates and floor areas (122,256 listings) were excluded. We also excluded listings from Nerima Ward (2,091 listings) to avoid multicollinearity among the dummy variables. Furthermore, listings with a floor area under 15 m² or over 150 m² were removed to align them with the scale of the STR units. Locational attributes—including distance to the nearest station (m) [32], station ridership (passengers/day) [33], and the number of tourist attractions within 1 km, 5 km, and 10 km [34] —were derived using GIS and the National Land Numerical Information database. Consequently, the final LTRDS contained 20,930 properties.

Using the STRHPM developed in Objective 1, we applied the property attributes of the LTRDS to estimate nightly rates and constructed the Converted Long-Term Rental Dataset (CLTRDS). This comprised 20,930 properties, which represented the status after conversion to STRs. Based on this dataset, we calculated the monthly total STR revenue (STRP, in JPY) and estimated the profitability ratio (PR) by dividing STRP by the original long-term rental price (LTRP, in JPY/month). This PR value, representing the ratio of the STR revenue after conversion to the LTR rent before conversion, was added to the CLTRDS. Because the Private Lodging Business Act in Japan limits the maximum number of operating days for STRs to 180 days per year (Japan Tourism Agency, 2018), we assumed a maximum of 15 operating days (OD) per month [37]. Airbnb occupancy rates in Tokyo are reported to be 93% or higher for the top 10% of listings, 87% or higher for the top 25% of listings, and around 71% for typical listings [38]. Based on these actual occupancy rates, we assumed three patterns of occupancy rates, from 60% to 80%, centered around 70%. The actual number of OD was calculated as 15 days multiplied by the assumed occupancy rates (OC) of 60%, 70%, and 80%. Following Airbnb’s regulations, we applied a 3% service charge (SC) which was deducted from the nightly rate.[39] Cleaning fees (CF) were incorporated, assuming a minimum two-night stay and that cleaning occurred once every two operating days (i.e., OD/2 times). However, this assumption may overstate cleaning costs. Cleaning fees were set based on the typical rates charged by STR cleaning service providers in Tokyo, as shown in Table 2. Accordingly, the monthly STR revenue (STRP) and PR was calculated using the following formulas (Equations 3 and 4):


$$STRP=\;EF\;\times \;OD\;-SC-\;CF\;\times \;\frac{OD}{\text{2}}\;$$ (3)

OD=15×OC

SC=EF×WD×3%

$$PR=\;\frac{STRP}{LTRP}\;$$ (4)

*EF*: Estimated nightly rate (JPY)

*OD*: Number of operating days per month

*SC*: Service Fee (JPY)

*CF*: Cleaning fee (JPY)

*OC*: Occupancy Rate

3%: Airbnb host service fee

To examine the property attributes associated with profitability, we constructed three bivariate logistic regression models using the CLTRDS, corresponding to the three assumed occupancy rates. The dependent variable was defined as a PR ≥ 1. The independent variables were categorized monthly rent (JPY/month), floor area (m²), straight-line distance to the nearest station (m), station ridership (passengers/day), presence of tourist attractions within a 1 km radius (binary), number of tourist attractions within 5 km and 10 km, and administrative district (excluding Nerima Ward). Unadjusted odds ratios (ORs) were calculated for each property attribute category to assess their correlation with the profitability ratio (Table 3).

### Objective 3: Construction and comparison of hedonic pricing models for LTR and STR profitability

To identify the property attributes that contribute to profitability for both LTRs and STRs, we developed two hedonic pricing models:

LTRHPM (Long-Term Rental Hedonic Pricing Model) estimated monthly rent for LTRs.

CLTRHPM (Converted Long-Term Rental Hedonic Pricing Model) estimated the profitability ratio (STRP/LTRP) after conversion.

The LTRHPM was developed using the LTRDS and specified monthly rent (JPY) as the dependent variable. Independent variables included floor area (m²), straight-line distance to the nearest station (m), station ridership (passengers/day), presence of tourist attractions within 1 km, number of attractions within 5 km and 10 km, and administrative districts (excluding Nerima and Edogawa wards).

The CLTRHPM used the profitability ratio as the dependent variable, and the same set of independent variables.

We compared standardized regression coefficients (β) and contribution rates (CR) between the two models to identify the property attributes that contributed to LTR profitability and STR profitability (see Equation 5 and Table 4).


$$CR=\frac{\beta^{\text{2}}}{\sum \beta^{\text{2}}}\times \text{100 }$$ (5)

*CR* = Contribution rate (%)

*β* = Standardized regression coefficient

## Results

### Objective 1: Construction of the hedonic pricing model for STR nightly rates

Table 1 presents the results of the multiple linear regression model (STRHPM) used to estimate STR nightly rates. The model was statistically significant and demonstrated moderate explanatory power (F-statistic = 215.6, p < .001; adjusted R² = 0.43). The variance inflation factors (VIFs) for all independent variables, excluding administrative district dummies, were below 10, suggesting a low risk of multicollinearity.[40]

The resulting model is expressed by the following linear equation, based on Equation (2):


NR=211.55·EA−2.76·DS+0.0068·PS+970.44·SS1+61.11·SS5+94.89·SS10+AD+1774.69             (6)

*NR*: Nightly rate (JPY/day)

The regression results indicated the following:

Estimated floor area (EA) had a strong positive association with nightly rates (β = 0.560), contributing significantly to price estimation.

Distance to the nearest station (DS) was negatively associated with nightly rates (β = –0.075), indicating that better accessibility increases STR value.

Station ridership (PS) had a positive impact on rates (β = 0.069), suggesting a preference for properties near busy transport hubs.

Presence of tourist attractions within 1 km (SS1) and the number of attractions within 5 km (SS5) and 10 km (SS10) were all positively and significantly associated with higher nightly rates (β = 0.104, 0.050, and 0.116, respectively), supporting the importance of local amenities and tourist appeal.

Several administrative districts such as Chiyoda, Minato, Shibuya, Meguro, and Setagaya were positively associated with higher nightly rates.

These results confirm that physical and locational property attributes—including size, accessibility, and proximity to tourist infrastructure—are significant determinants of STR pricing in Tokyo.

### Objective 2: Logistic regression models and odds ratio analysis for PR ≥ 1 across three occupancy scenarios

Table 3 summarizes the results of the three bivariate logistic regression models predicting whether PR is greater than or equal to 1 under the assumed STR occupancy rates of 80%, 70%, and 60%. The following trends were observed.

Monthly rent: Properties in the ¥100,000–¥200,000 range showed significantly lower odds of achieving PR ≥ 1 at both 70% and 60% occupancy. This suggests that lower-rent properties are more likely to benefit from the STR conversion.

Floor area: Units in the 35–55 m² category had significantly lower odds at all three occupancy levels (80%: OR = 0.81; 70%: OR = 0.50; 60%: OR = 0.32), indicating that larger units are more favorable for STR profitability, especially under lower occupancy conditions.

Distance to the nearest station: Longer distances were associated with significantly lower odds. Properties within 500 m of a station consistently outperformed those farther away, confirming that station proximity is a key factor in STR profitability.

Station ridership: Properties near stations with 200,000–300,000 daily passengers had the highest odds of profitability (80%: OR = 13.80; 70%: OR = 14.60; 60%: OR = 15.06), underscoring the importance of major transit hub accessibility.

Tourist attractions: The absence of attractions within 1 km significantly decreased odds (80%: OR = 0.08; 70%: OR = 0.09; 60%: OR = 0.07). A higher number of attractions within 5 km and 10 km was positively and significantly associated with profitability at all occupancy levels.

Administrative districts: Wards such as Shibuya, Meguro, and Shinjuku exhibited very high odds ratios at 80% occupancy (e.g., Shibuya: OR = 88.30), reflecting their status as major transport and commercial hubs. Conversely, wards such as Katsushika, Ota, and Kita displayed significantly lower odds (e.g., Katsushika: OR = 0.02), suggesting that STR conversions in these locations are less likely to be profitable.

STR profitability depends strongly on accessibility, surrounding amenities, and district-specific characteristics.

### Objective 3: Comparison of hedonic pricing models for LTR and STR profitability

Table 4 presents the results of two hedonic pricing models: LTRHPM, which estimates the monthly rent for LTRs, and CLTRHPM, which estimates the profitability ratio (STRP/LTRP) following conversion to STR.

Findings from the LTRHPM

Among the property attributes contributing more than 1% to LTR rent:

Floor area contributed overwhelmingly (CR = 80.0%, β = 0.824)

Number of tourist attractions within 10 km (CR = 1.84%, β = 0.13)

Distance to the nearest station (CR = 1.45%, β = –0.111)

Administrative districts such as Setagaya (CR = 4.53%, β = 0.20), Suginami (2.41%), Meguro (2.34%), Ota (1.75%), Shibuya (1.18%), Minato (1.11%), and Adachi (1.04%) also exhibited positive contributions, except for Adachi, which had a negative association (β = –0.094).

These results suggest that LTR profitability is driven primarily by property size and secondarily by locational advantages in specific wards.

Findings from the CLTRHPM

In the model estimating the post-conversion profitability ratio, the following attributes contributed more than 1%:

Distance to the nearest station (CR = 15.79%, β = –0.355)

Number of tourist attractions within 10 km (11.43%, β = 0.30)

Floor area (5.32%, β = 0.21)

Station ridership (3.01%, β = 0.16)

**Fig 2:**
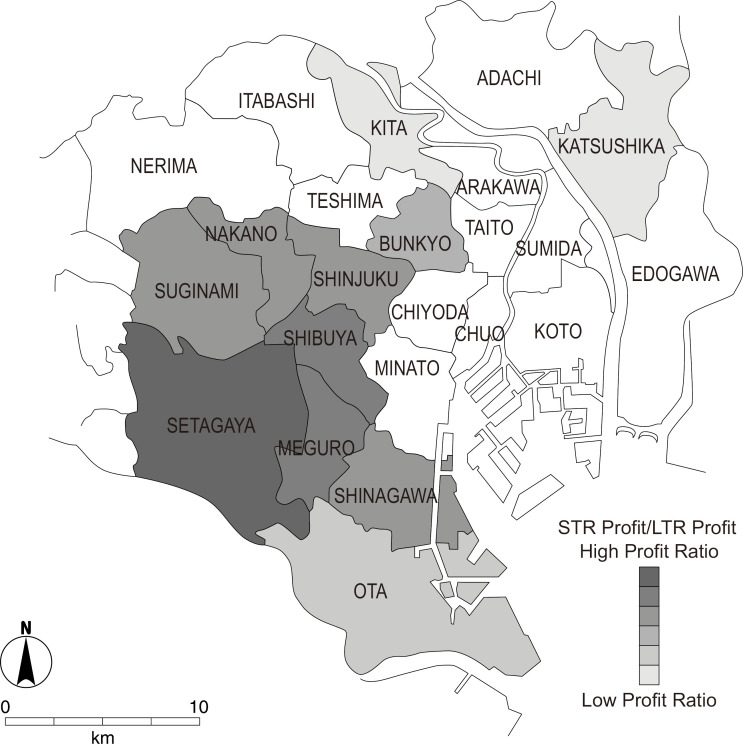
Administrative districts contributing more to STR profitability than to LTR (dark) and those contributing more to LTR profitability than to STR (light). *Source:* National Land Numerical Information (Administrative Boundaries Data), Ministry of Land, Infrastructure, Transport and Tourism of Japan (MLIT), which is free to use under Article 13 of the Japanese Copyright Act (public domain).

Administrative districts with significant positive contributions included (Fig 2):

Setagaya (27.91%, β = 0.47)

Shibuya (7.96%, β = 0.25)

Meguro (6.23%, β = 0.22)

Suginami (3.66%, β = 0.17)

Nakano (2.56%, β = 0.14)

Shinagawa (1.36%, β = 0.10)

Shinjuku (1.08%, β = 0.19)

In contrast, negative associations were observed for:

Distance to the nearest station (β = –0.355)

Katsushika (β = –0.20)

Kita (β = –0.17)

Ota (β = –0.11)

These results indicate that STR profitability is more heavily influenced by proximity to transit hubs and tourism density than property size alone. In contrast to LTRs, where floor area dominates, STRs benefit more from location-based advantages.

## Discussion

The main findings of this study indicate that certain property attributes contribute significantly to increased profitability when vacant LTRs are converted into STRs. These include a shorter distance to the nearest station, a higher number of tourist attractions within 10 km, a larger floor area, greater station ridership, and locations in specific administrative districts, such as Setagaya, Shibuya, and Meguro (Table 4). Although floor area contributed overwhelmingly to LTR rent (80%), it played a comparatively smaller role in STR profitability (5.3%). Conversely, attributes such as proximity to stations and tourist attractions made significantly higher contributions to STR profitability than LTR rent, highlighting the asymmetric influence of property attributes on the two types of rentals.

### Property characteristics

Bivariate logistic regression models for three assumed occupancy rates indicated that properties with lower monthly rent were more likely to achieve profitability (PR ≥ 1) after STR conversion (Table 3). This suggests that high-rent units are less favorable for conversion, whereas lower-rent units have a higher potential for revenue gain through STR operations.

Thus, the key property characteristic contributing to LTR profitability is a larger floor area, whereas a lower pre-conversion rent is advantageous for STR profitability. Larger units with higher rents tend to be more favorable in the long-term rental market, where spacious living and functional layouts are valued by long-term occupants, particularly families. In contrast, short-term guests are less sensitive to interior spaciousness and are more concerned with convenient, functional locations for temporary stays.

### Locational attributes

A shorter distance to the nearest station, which is a key factor in STR profitability, indicates that accessibility to public transportation significantly enhances nightly rates. Properties located within 500 m of a station (approximately a 6-minute walk) had the highest odds of STR profitability. Although station proximity is also valued in the rental market, it is especially critical for travelers carrying luggage and seeking access to multiple destinations within a limited timeframe. This finding supports prior research identifying transport accessibility as a determinant of STR pricing [41].

Similarly, stations with a ridership between 200,000 and 300,000 passengers per day—classified as urban transit hubs in Japan—were associated with the highest STR profitability. These hubs offer connections to multiple train lines and are often surrounded by dense commercial and retail activities, which enhance the convenience for short-term guests. However, these areas may be less desirable for long-term tenants because of congestion, noise, or a lack of residential tranquility.

The positive impact of nearby tourist attractions, especially those within 10 km, also aligns with guest preferences. Given that the average distance between stations on Tokyo’s Yamanote Line is approximately 1.2 km [42], access to a wide range of tourist destinations within a few stops is advantageous for STR locations. In contrast, proximity to tourist sites had a limited influence on LTR rents, highlighting the differing locational preferences between residents and visitors. This finding reinforces earlier studies emphasizing the importance of tourism-oriented location factors in STR pricing [43–46].

Wards with the highest STR profitability—such as Shinjuku (2.98 million daily passengers), Shinagawa (750,000), Meguro (640,000), and Shibuya (2.80 million)—all host major regional transport nodes, supporting the notion that transit accessibility and commercial density are key drivers of STR value. Although more peripheral, Nakano and Suginami also offer good access while maintaining lower real estate prices, which aligns with the finding that low-rent areas may offer high STR returns.

By contrast, areas with high LTR profitability, such as Minato Ward and Setagaya, tend to cater to long-term, high-quality residential demand because of their proximity to central business districts and superior living environments. These results suggest that, while the STR and LTR markets physically overlap, their value drivers differ based on user needs and market structure. The findings further reinforce prior studies in Western contexts that show contrasting valuation mechanisms between the STR and LTR markets [44,47].

### Limitations

This study had several limitations. It focused exclusively on Tokyo’s 23 wards. Applying these findings to other cities should consider local variations in tourism demand, housing markets, and STR regulations. The Airbnb dataset used was from September 5, 2024 but STR markets are fluid and subject to seasonal and policy-driven changes [48]. Events such as the COVID-19 pandemic and the 2020 Tokyo Olympics were not considered. Dogru et al. reported that Airbnb supply is affected by macroeconomic factors [49]. We also excluded STR-listing features such as host profile, amenities, and review ratings. While this study focused on structural and locational attributes, previous studies revealed that host characteristics and listing quality significantly influence STR pricing [50–55]. Furthermore, we did not include local STR regulations such as ward-level ordinances [56]. Specifically, in wards other than Sumida, Toshima, Kita, Katsushika, and Edogawa, the profitability of the model may be overestimated due to certain regulations regarding business days and zoning of city planning. We also excluded outliers using a Z-score filter (–2 ≤ Z ≤ 2), which may have reduced the impact of extremely high- or low-priced listings. In addition, assumptions regarding occupancy rates, cleaning costs, and service fees were applied uniformly, which may not reflect operational variability. It should be emphasized that the revenue data in this study are not observational, but are based on open data and various plausible assumptions, which limits the generalizability of the findings. Despite these limitations, this study offers meaningful insights into the distinct drivers of STR and LTR profitability and provides a basis for future research using longitudinal or multi-city comparative approaches.

## Conclusions

This study examined the profitability of converting vacant LTRs into STRs in Tokyo’s 23 wards using open datasets and hedonic pricing models. By comparing the contributions of various property attributes to the LTR rent and STR profitability, we found that the two rental types are influenced by distinct value drivers. While floor area is the dominant factor in LTR rent, STR profitability is more strongly associated with proximity to stations, station ridership, and access to tourist attractions. Properties located in well-connected areas with lower real estate prices, such as Nakano and Suginami, were found to offer high profitability when converted to STRs, although they may be less favorable in the LTR market. These findings provide evidence-based insights into the potential of vacant rental housing stock to meet the growing demand for tourist accommodations. They also contribute to discussions on rational STR regulations and urban housing utilization strategies, especially in cities that balance vacancy problems with tourism-driven demand.
